# Safety Profile of Monoclonal Antibodies and Subsequent Drug Developments in the Treatment of Paroxysmal Nocturnal Hemoglobinuria

**DOI:** 10.3390/medicina60030379

**Published:** 2024-02-24

**Authors:** Vasantha Mallenahalli Neeekantappa, Ashwin Kamath, Poovizhi Bharathi Rajaduraivelpandian

**Affiliations:** Department of Pharmacology, Kasturba Medical College, Mangalore, Manipal Academy of Higher Education, Manipal, India; vasantha.mn@manipal.edu (V.M.N.);

**Keywords:** paroxysmal nocturnal hemoglobinuria, eculizumab, ravulizumab, adverse drug reaction, challenges, investigational new drugs

## Abstract

Paroxysmal nocturnal hemoglobinuria (PNH) is a clonal stem cell disease characterized by intravascular hemolysis due to the targeting of affected red blood cells by the complement system. Eculizumab and ravulizumab are two monoclonal antibodies that inhibit the complement system’s components and have been shown to significantly improve survival and quality of life. This review describes the role of these monoclonal antibodies in the treatment of PNH with an emphasis on their safety profile. The challenges in the use of these drugs and new drugs in various stages of drug development are also described, which may be helpful in addressing some of these challenges.

## 1. Introduction

Paroxysmal nocturnal hemoglobinuria (PNH) is a rare clonal stem cell disease [1]. It is characterized by the ability of the complement system to target blood cells that are deficient in some surface proteins, including the two complement regulators CD55 and CD59, and cause the creation of extracellular vesicles [2]. Clinically, hemoglobinuria, exhaustion and shortness of breath are the most common symptoms; other manifestations include intravascular hemolysis, thrombosis and bone marrow failure [3].

Phosphatidylinositol glycan class A (*PIG-A*) gene mutations, an X-linked gene that produces an enzyme required for the conversion of N-acetyl glucosamine to phosphatidylinositol, are connected to PNH [4,5]. Glycosyl phosphatidyl inositol (GPI) anchor biosynthesis starts with this step. The dysfunction of this metabolic process prevents all GPI-linked proteins from being expressed on the surface of afflicted cells [5]. The production of a sizable proportion of progeny blood cells with the PNH phenotype results in the typical triad of intravascular hemolysis, thrombophilia and bone marrow failure. With excellent sensitivity and specificity, flow cytometry can be used to detect a number of GPI-anchored proteins, most notably CD55 and CD59 [6]. In patients with PNH receiving eculizumab therapy, hemolytic complement (CH50) activity indicates C5 blockage and is directly correlated with levels of circulating free eculizumab. Therefore, CH50 monitoring is necessary [7].

Approximately five decades ago, the 10-year survival rate in patients with PNH was just 50%; however, over the past 15 years, the development of monoclonal antibodies has increased survival to more than 75% [8]. In this review, we discuss the role of monoclonal antibodies in the treatment of PNH with a specific focus on the safety of two commonly used drugs, eculizumab and ravulizumab. We also describe newer therapeutic options. The search strategy used to identify the relevant studies is shown in Supplementary File S1.

## 2. Treatment of PNH

**Figure 1:**
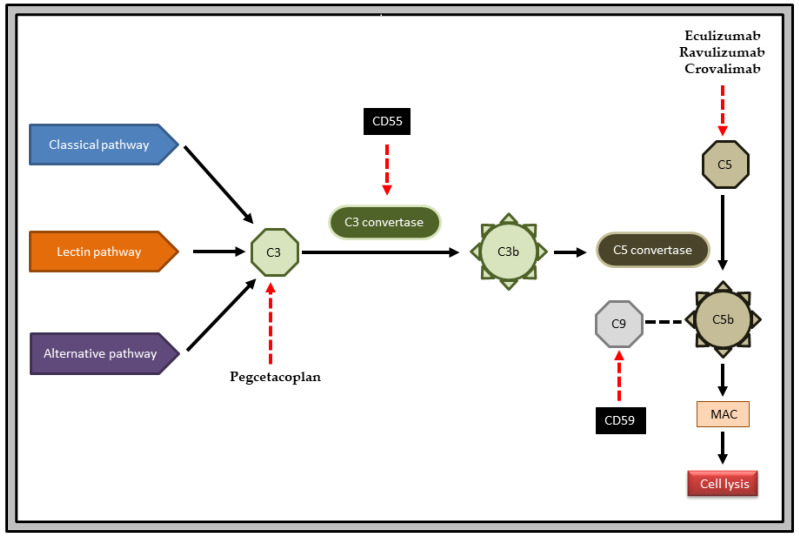
PNH pathogenesis and mechanism of action of important drugs. The complement cascade is initiated by three different pathways: classical, lectin and alternative. Conversion of C3 to C3b is mediated by C3 convertase, and that of C5 to C5b is mediated by C5 convertase. C5b enables the formation of membrane attack complex (MAC) and leads to cell lysis. CD55 inhibits the enzyme C3 convertase. CD59 inhibits the incorporation of C9 into MAC. In PNH, there is a deficiency of CD55 and CD59. The anti-C5 drugs are eculizumab, ravulizumab and crovalimab. The anti-C3 drug is pegcetacoplan.

The development of the anti-C5 monoclonal antibodies eculizumab and ravulizumab has significantly improved the survival rate in patients with PNH [9] (Figure 1). Besides the use of monoclonal antibodies or newer small molecules such as pegcetacoplan, patients with PNH frequently require supplementation with iron, folic acid and vitamin B_12_ [10]. High doses of erythropoietin or darbepoetin are used for impaired erythropoiesis, and G-CSF is used for granulocytopenia [11,12]. Anticoagulants such as warfarin or acenocoumarol are used for acute thrombotic episodes or clinically severe thrombosis. In pregnancy, low-molecular-weight heparin should be administered [13]. A thrombolytic is used to treat venous thrombosis [14]. Glucocorticoids, such as prednisone 0.3–0.6 mg/kg/day, are used to treat extravascular hemolysis [15,16]. Antioxidants are useful as adjuvants [17]. Patients with severe aplastic anemia with PNH clones who do not respond to eculizumab therapy because of heterozygous c.2654G-A mutations in C5 and those who are young and have a potential donor are strong candidates for bone marrow transplantation [18]. To eliminate the PNH clone, non-myeloablative conditioning treatment is necessary. A high dose of cyclophosphamide administered post-transplant may be particularly helpful in PNH [19].

## 3. Role of Monoclonal Antibodies

Eculizumab is a humanized monoclonal antibody that binds to the human C5 complement protein with high affinity, inhibiting its cleavage into C5a and C5b and preventing the formation of the terminal complement complex C5b-9 (membrane attack complex), which is responsible for the lysis of PNH RBCs lacking the cell surface CD59 [20,21,22]. Eculizumab has a molecular weight of approximately 148 kDa and is made up of two 448 amino acid heavy chains and two 214 amino acid light chains [23]. It binds to human tissues such as smooth and striated muscle as well as the renal proximal tubular epithelium. Eculizumab is metabolized by lysosomal enzymes to small peptides and amino acids. Its volume of distribution in humans is similar to that of plasma [24].

Ravulizumab varies from eculizumab by substituting four amino acids, which changes the pharmacokinetics and pharmacodynamics of the molecule [25]. Similar in safety and tolerability to eculizumab, ravulizumab has some advantages. Ravulizumab is administered intravenously every eight weeks and has a four-time-longer half-life than that of eculizumab, with dosage intervals of up to 12 weeks; it is also used to treat cancer. The dosage strategy is based on body weight [26].

## 4. Clinical Evidence Supporting the Efficacy of Eculizumab and Ravulizumab in PNH

Several studies have shown the usefulness of eculizumab for treating PNH (Table 1). Regarding ravulizumab, dosing regimens that result in a higher trough concentration of the drug increase the frequency of LDH normalization and decrease breakthrough hemolysis events [27]. Ravulizumab reduces complement-mediated inflammatory damage associated with COVID-19 infection, particularly in patients with PNH [28].

## 5. Safety of Eculizumab

### 5.1. Broad Outlook

The development of eculizumab has provided a targeted, disease-modifying treatment that is well tolerated and reduces the risk of hemolysis, fatigue, anemia, transfusion requirements, renal failure, pulmonary hypertension and thromboembolic events. It can improve anemia and quality of life [10]. Eculizumab has become the therapeutic gold standard for hemolytic PNH patients and has significantly improved survival [35]. Eculizumab prevents premature birth, one of the most devastating complications in pregnancy, and reduces the risk of chronic kidney disease and cardiovascular disease in the offspring of pregnant patients with PNH. The dose is 1200 mg every second week during the third trimester until delivery. A small amount is excreted in breast milk, but breastfeeding is safe [35,36].

The safety of eculizumab in patients with PNH was evaluated in seven studies [1,2,3,6,7,9,10], with the number of patients per study ranging between 11 and 195. This low rate of recruitment can be attributed to the rarity of PNH, which is predicted to occur in 15.9 people per million people worldwide [14]. However, some researchers attribute these numbers to underdiagnosis in people with minimal manifestations or in those with concurrent illnesses, which makes it difficult to arrive at a diagnosis [19]. The study duration ranged from 16 to 64 weeks. Kanakura Y et al. and Hill A et al., in their studies, had an initial study period of 12 weeks each and an extension study period of 24 and 52 weeks, respectively [2,7]. The extension studies enabled evaluations of long-term tolerability, safety and efficacy given that safety issues can either be extremely uncommon or have an extended latency time and might not be quantifiable over a short duration [37]. Pharmacovigilance analysis reported a cumulative exposure to eculizumab of 21,016 patient years for a period of 10 years [9]. Because the number of participants and duration of the study varied widely, the safety parameters are compared in percentages in this review. The reported percentage of PNH patients who developed adverse events ranged from 10% to 96% [2,3,10]. This can be explained by the longer study duration in Hillmen P et al.’s 2013 study (up to 52 weeks) when compared to the other two studies, those of Kanakura Y et al. (up to 24 weeks) and Hillmen P et al. 2021 (up to 16 weeks), which means the incidence of adverse events might reduce in long-term treatment with eculizumab compared to short-term treatment [2,3,10]. During the 52-week period, there were 19 treatment withdrawals and 1 death among the 195 trial participants across seven studies [3]. Death was due to chronic myeloid leukemia, and treatment withdrawals were due to nonfatal adverse events, pregnancy, myelodysplastic syndrome, meningococcal sepsis, worsening of PNH and thrombotic events. The frequency range of eculizumab adverse effects is presented in Table 2. Overall, eculizumab was found to be well tolerated. A system-wise brief description of the adverse events is presented below.

### 5.2. Disorders of the Musculoskeletal and Nervous System

Headache was the most frequently reported adverse event in up to 54.9% of the study participants [3]. Most occurred 24 h after the study medication was administered. An immediate decline in the hemolysis brought on by eculizumab can cause an abrupt surge in levels of nitric oxide, resulting in an initial spike in the frequency of headaches [38,39,40]. Similarly, the decrease in headaches after the first two doses of eculizumab may represent a physiological restoration to steady-state levels of nitric oxide [41].

Insomnia and fatigue were reported in up to 20.5% and among 15% of participants, respectively. This is in contrast to the findings of a study that reported significant improvement in the quality of life domains, insomnia (*p* = 0.03) and fatigue (*p* < 0.001), rapidly and for extended periods of study [7]. Lumbar/sacral disc prolapse was reported in one participant (2%) in one out of the seven studies.

### 5.3. Disorders of the Digestive System

Nausea and vomiting were reported in up to 32.3% and 25.6% of the participants, respectively, receiving eculizumab [3]. Compared with constipation (14.9%), diarrhea (34.9%) was encountered more commonly [3].

### 5.4. Disorders of the Thoracic, Respiratory System and Mediastinum

Pharyngitis, nasopharyngitis, upper respiratory tract infection, oropharyngeal pain, pneumonia, flu-like symptoms, cough, influenza-like illness and sinusitis were reported in up to 36.4%, 77.8%, 41%, 21.5%, 14.8%, 36.4%, 27.2%, 17.4% and 11.8% of participants, respectively [2,3,7]. There does not appear to be a specific correlation between the timing of infections and the duration of therapy or exposure. This could be because, apart from encapsulated bacteria, such as *Streptococcus pneumoniae* and *Neisseria meningitidis*, a terminal complement plays a lesser role in the defensive mechanism [35].

### 5.5. Disorders of the Blood, Vascular and Lymphatic System

Anemia and hemolysis are observed in up to 14.8% and 23% of participants, respectively, most frequently concurrent with an infection [3]. Contusion and epistaxis are observed in up to 27.2% and 11.1% of participants, respectively. The ability of eculizumab to lower d-dimer and F1 + 2 levels suggests that it inhibits C5a and C5b-9 production and bioactivity through terminal complement inhibition, leading to coagulation cascade downregulation [42]. Thrombotic events, which are potentially fatal, were reported in only up to 2% of participants with PNH [1]. However, a minimum of a single thromboembolic crisis occurs in one-third of patients with PNH over the course of the illness. Since the initial use of eculizumab, there has been a significant decrease in the frequency of thromboembolic complications [28]. Among several studies, myelodysplastic syndrome (in 1.54% of participants) was discussed in only one study [3]. There is considerable overlap seen between PNH and MDS, and up to 5% of patients with MDS have PNH cells that lack glycophosphatidylinositol [36].

### 5.6. Immunology

Pruritus, rash, injection site pain and drug-infusion-related events were reported in up to 10.3%, 10.3%, 6.3% and 36.4% of participants, respectively. None of the adverse events linked to medication infusion led to the discontinuation of treatment [3]. Antibodies were not detected in any of the participants. Human antihuman antibodies (HAHAs) were only rarely and transiently observed in clinical studies of eculizumab in patients with PNH, with no effect on the clinical outcome. After a follow-up of more than 66 months, only 5 out of 161 patients (3.1%) experienced brief positive responses on HAHA tests [43].

### 5.7. Malignancy

Chronic myelomonocytic leukemia was the cause of death in one patient with MDS and PNH [3]. A pharmaceutical-company-maintained pharmacovigilance database contains reports of malignancies in patients with PNH (2.6 reports per 100 person-years). Skin neoplasms, which account for 15% of solid tumors, were observed in patients with PNH [9]. No other studies have reported an association between eculizumab therapy and malignancy.

### 5.8. Pregnancy

Eculizumab was administered to two pregnant women during the first 4 and 5 weeks of pregnancy. Both pregnancies were event-free, and both infants were born healthy and without any side effects [3]. A pharmaceutical-company-maintained pharmacovigilance database also has reports of 335 pregnant individuals with PNH who were exposed to eculizumab. Of pregnancies with recorded outcomes, 72.8% ended in live births; miscarriages, induced abortion, stillbirths and maternal deaths accounted for the rest [9]. Blood from umbilical cord samples contains insufficient eculizumab levels to affect a newborn’s complement concentrations. Therefore, eculizumab may be considered safe during pregnancy [44].

### 5.9. Infection

Urinary tract infection, meningococcal infection, pyrexia and staphylococcal infection were reported in up to 16.9%, 1%, 11.8% and 2% of the participants, respectively. For eculizumab therapy, this may be a limiting factor. Eculizumab increases the risk of infection because it inhibits complement effector pathways (C5-C9). In particular, *Neisseria meningitides* and pyogenic and encapsulated bacteria are important in this regard [45]. Viral infection was reported in up to 92.6% of participants. This is because B lymphocyte enhancement, which prevents virus-induced diseases by complement-mediation, is inhibited by eculizumab [46]. Aspergillus infection was reported in up to 3% of participants [3]. This is because the fungi are resistant to MAC-mediated lysis because of their robust cell walls. In addition to opsonization with the fragments derived from C3 for effective phagocyte binding, complement actions against Aspergillus also result in the development of an inflammatory response with C5a, an anaphylatoxin [47,48]. It is possible that the functional C5 deficiency linked to eculizumab use was a factor in the emergence of Aspergillus infection.

## 6. Safety of Ravulizumab versus Eculizumab

### 6.1. Broad Outlook

Ravulizumab is a long-lasting recycled IgG monoclonal antibody with a higher affinity for the neonatal Fc receptor (FcRn). IgG homeostasis regulation requires FcRn [49]. High IgG doses cause the FcRn pathway to become saturated, as they compete with endogenous IgG for binding to FcRn via their Fc regions, leading to increased IgG clearance [50].

IgG concentrations were tracked over time in adult patients with PNH receiving ravulizumab, demonstrating that levels of IgG and IgG subclasses controlled by FcRn were unchanged [49]. Therefore, in these individuals, there is no concern regarding treatment-related hypogammaglobulinemia increasing the risk of infection complications or leading to a recurrence of PNH-related symptoms or breakthrough hemolysis [19].

The safety of ravulizumab and eculizumab in patients with PNH patients was compared in six studies [27,51,52,53,54,55], with significant differences in the study design, duration and number of participants. Headaches were the most frequent adverse drug reaction noticed in both ravulizumab and eculizumab groups in all the studies, followed by upper respiratory tract infection, nasopharyngitis and pyrexia. The incidence of treatment-emergent adverse events (TEAEs) was lower in the extension period than that in the period of primary evaluation in both groups [52,53]. The serious adverse event (SAE) percentage reduced in the arm, where study participants were shifted from initial eculizumab therapy to later ravulizumab therapy, which may be beneficial to patients [52,53,54]. The frequency range of ravulizumab and eculizumab adverse effects is presented in Table 3. The overall safety profile of both drugs was found to be similar. Most of the adverse effects were found to be lesser during the extension period, when participants continued ravulizumab therapy or when they were shifted from eculizumab to ravulizumab therapy.

### 6.2. Disorders of the Musculoskeletal and Nervous System

The frequency of headaches was similar in both ravulizumab (up to 36%) and eculizumab (up to 33.1%) -treated study participants during the primary evaluation period [52]. There was a greater drop in the percentage of headaches among ravulizumab (up to 6%) -treated participants than that among eculizumab (up to 10.5%) -treated participants during the extension period [52,54]. Musculoskeletal pain, arthralgia, back pain, myalgia and insomnia were more frequent among eculizumab-treated participants than those among ravulizumab-treated participants [51,52,53,54]. The frequency of pain in the extremities and dizziness were found to be similar or higher in ravulizumab-treated participants than in those treated with eculizumab [51,52,53,54].

### 6.3. Disorders of the Digestive System

Abdominal pain and dyspepsia were more frequent in eculizumab-treated participants [27,52,53]. Vomiting, constipation and diarrhea were more frequent in ravulizumab-treated participants [51,52,53,54]. The frequency of nausea varied in the studies with both treatments [27,51,52,53].

### 6.4. Disorders of the Thoracic, Respiratory System and Mediastinum

Nasopharyngitis, rhinitis and upper respiratory infections were found to be more frequent in ravulizumab-treated participants [51,52,53,54]. Oropharyngeal pain, cough, influenza-like illness and viral upper respiratory infections were more frequent in eculizumab-treated participants [51,52,53].

### 6.5. Disorders of the Blood, Vascular and Lymphatic System

TEAEs that were considered major adverse vascular events and anemia were found more frequently in ravulizumab-treated participants [27,52,53,54]. Chest pain and hypokalemia were more frequent in eculizumab-treated participants [52,53].

### 6.6. Infection and Immunology

Influenza and lower respiratory tract infection, both considered serious by the investigators, were diagnosed in two ravulizumab-treated participants [51,53]. Acute pyelonephritis was diagnosed in one of the eculizumab-treated participants [53]. Pyrexia was more frequent in eculizumab-treated participants [51,52,53,54]. Infusion site reactions occurred more frequently in ravulizumab-treated participants [27,55]. Antidrug antibodies were found at similar frequencies in both drug therapies [27,51,52,53,54].

## 7. Difficulties/Challenges in Using MABs in PNH

Despite being effective in treating PNH, eculizumab therapy has certain drawbacks [3]. Additionally, as eculizumab has an 11-day half-life, it must be administered intravenously perpetually every 2 weeks [56]. It was demonstrated that individuals with PNH have psychosocial pressure as a result of this dose regimen, which affects both their capacity to perform at work and their relationships with friends and family [57]. In addition, 25–35% of patients on eculizumab treatment still need red blood cell transfusions because of breakthrough hemolysis, opson-mediated extravascular hemolysis and bone marrow loss [58].

The frequent and indefinite need for eculizumab dosing also contributes to the high cost of treatment. Based on the cost-effectiveness analysis study conducted in the United States in 2017 and Canada in 2014, the authors concluded that, for eculizumab to be cost-effective for the treatment of PNH, its price would need to be cut by 98.5% [15].

In addition, although eculizumab is beneficial in most PNH patients, a small subset of individuals (3.2%) was shown to carry a single missense mutation on C5 that prevents eculizumab binding and causes a subpar therapeutic response [59].

### 7.1. Breakthrough Hemolysis (BTH)

BTH was reported with eculizumab, which decreased after switching to ravulizumab [60]. Either pharmacokinetics or pharmacodynamics underlie BTH caused by C5 inhibition. Pharmacokinetic BTH is triggered by insufficient drug levels, which result in inadequate C5 inhibition (free C5 ≥ 0.5 µg/mL); this typically occurs 10 days or more after the last dose of medication and tends to occur frequently [58]. Pharmacodynamic BTH occurs even when medication levels are sufficient. Eculizumab and ravulizumab do not prevent C5 from adopting a C5b-like conformation under conditions of high complement activation, such as major surgery, the third trimester of pregnancy and infection, in PNH red cells that are densely coated with C3b [61,62]. However, with ravulizumab, pharmacokinetic BTH occurs less frequently. Between the two medications, there is no difference in pharmacodynamic BTH [63].

### 7.2. Meningococcal Infection

Meningococcal illness is detected 1000–2000 times more frequently in patients who use eculizumab [64]. Between 2008 and 2016, there were 16 occurrences of meningococcal illness in eculizumab recipients in the US; 11 of these cases were caused by nongroupable *Neisseria meningitidis*. Fourteen patients presented proof that they had received at least one dose of meningococcal vaccination before the development of symptoms [64]. Some healthcare professionals in the United States and public health organizations in other nations advise antimicrobial prophylaxis for the entire duration of eculizumab treatment in recipients, as they are still at risk even after receiving meningococcal vaccines; for many patients, a lifelong treatment is anticipated [64]. Chemoprophylaxis with penicillin is advised; those with penicillin allergies are often advised to take macrolides [65]. Regardless of meningococcal vaccination or chemoprophylaxis status, all patients undergoing eculizumab therapy must maintain increased vigilance, seek care early and treat any symptoms resembling meningococcal illness quickly. In 10 of the reported instances, meningococcemia was present but not meningitis. Meningococcemia is characterized by a petechial or purpuric rash, but this rash may not manifest until much later in the course of the illness [64]. Meningococcemia may start with relatively nondescript, mild-to-moderate initial symptoms such as chills, fever, vomiting, fatigue, diarrhea and pain in the chest, joints, muscles or abdomen. However, within hours, these symptoms can worsen to the point of serious sickness and death [64].

### 7.3. Quality of Life (QoL) and Work Productivity

In a cross-sectional survey among participants aged 18 years and older who had self-reported PNH diagnoses and were being treated with either eculizumab or ravulizumab, compared with the mean FACIT-Fatigue scores reported for the average US population (43.6), those of participants on PNH treatment were lower (eculizumab, 29.3 ± 14.0; ravulizumab, 33.3 ± 13.0) [66,67]. On the EORTC QLQ-C30, participants receiving eculizumab and ravulizumab reported mean scores for global health status of 62.4 (±21.1) and 67.2 (±19.0), respectively, compared with an average population score of 75.7 [67,68]. Users of eculizumab and ravulizumab had physical functioning scores of 76.4 (±17.5) and 76.7 (±20.3), respectively, both of which were below the reported average for the overall population of 91.0 [67,68]. WPAI questionnaire findings showed that over 80% of survey respondents who were working reported decreased job productivity and general activity impairment. In addition, nearly half (47.2%) of the respondents said they had missed hours of work in the last seven days before the conclusion of the survey. This shows that many patients with PNH receiving eculizumab or ravulizumab endure a significant burden of illness, as indicated by self-disclosed clinical variables, PNH symptoms and psychometrically validated scales confirming decreased QoL, despite therapy [67]. These research findings highlight the need for more effective medications to treat PNH because they show that the effects of the disease on patients’ QoL are not entirely mitigated by the present treatment options [67].

### 7.4. Orphan Drug

Eculizumab is an orphan drug; inaccessibility and a high price restrict access to the drug. There are reports of eligible patients being unable to afford the high treatment costs [69] or experiencing disease decompensations from forced therapy reductions caused by the non-availability of the drug [70,71]. The pricing of orphan medications is often rather high [72,73]. The annual patient cost of eculizumab in the US was about USD 432,240 to 542,640 per patient based on estimations of annual utilization derived using the usual FDA-approved dose [74]. These costs are typically greater than those for other undesignated medications [75]. Eculizumab was one of the top 10 most expensive orphan medicines in the world in 2017 [76].

## 8. New Drugs Available or under Development

### 8.1. Pegcetacoplan, C3 Inhibitor

Pegcetacoplan is a novel medication for treating PNH. Hemolysis that occurs in both intravascular and extravascular compartments can be inhibited by this C3 inhibitor. It is delivered twice a week as a subcutaneous infusion [77]. In clinical studies, pegcetacoplan outperformed eculizumab in patients with PNH with hemoglobin < 10.5 g/dL despite prior eculizumab medication in terms of reducing transfusion reliance and fatigue reduction [10,77]. Adverse effects can include infections of the respiratory tract, diarrhea and headaches [10]. Castro et al. identified that two patients with PNH who received pegcetacoplan in the trial experienced eight severe TEAEs, which included three urinary tract infections, one instance of pyrexia, pancreatitis and a lower gastrointestinal hemorrhage [78]. If appropriately administered, pegcetacoplan should improve patients’ QoL by lowering the risk of potentially fatal consequences of the disease. However, no adequate survival data are currently available for this medication [79].

### 8.2. Iptacopan, a Complement Factor B Inhibitor

Iptacopan is a new, oral complement factor B selective inhibitor under clinical development for PNH [80]. In a 2-year study, at the point of the interim analysis by 12 weeks, 12 of 13 PNH patients evaluated for efficacy achieved the primary endpoint of ≥60% reduction in serum LDH levels. Mean LDH levels fell swiftly and significantly, and hemoglobin levels improved clinically significantly in most patients. All but one patient continued transfusion-free through week 12 [80]. Other markers for hemolysis, such as bilirubin, haptoglobin and reticulocytes, also showed steady improvements [80]. Iptacopan was well tolerated, and no SAEs were reported [80]. Recently, two phase III confirmatory trials were initiated [81,82]. Iptacopan may become the very first oral monotherapy complement inhibitor for adult patients with PNH once the remaining phase III trial is completed [80,83].

### 8.3. Crovalimab, a C5 Inhibitor

Crovalimab is in phase III clinical trials. The standard of treatment for PNH patients with substantial clinical symptoms is complement C5 inhibition. Drug development is hampered by the complete and ongoing inhibition of the terminal complement pathway as well as elevated blood C5 content, leading to intravenous-only therapy choices [84].

In contrast to intravenous medications, crovalimab, “a sequential monoclonal antibody recycling technology” antibody, was developed for extended self-administration of subcutaneous dosages under conditions favorable to C5 inhibition [84]. Both eculizumab and ravulizumab have reduced efficacy in patients with PNH with hereditary nonsynonymous single-nucleotide polymorphisms (SNPs) in C5α subunits altering Arg885 (c.2654G → A, c.2653C → T), which correspond to the targeted epitope; ≤3.5% of people of Asian origin have these polymorphisms [51,59]. However, crovalimab binds to and inhibits the function of both wild-type C5 and a number of C5 variants with nonsynonymous SNPs, such as Arg885 [85]. A three-part open-label adaptable phase I/II clinical trial was conducted to evaluate the safety, pharmacokinetics, exploratory efficacy and pharmacodynamics in healthy participants in part 1, in complement blockade-naive patients in part 2 and in patients with PNH receiving treatment with a C5 inhibitor in part 3. C5 levels and hemolytic function were reduced below clinically meaningful thresholds. Safety was in line with the profile of C5 inhibition that is well known. Drug–target–drug aggregates were seen in all 19 individuals shifting to crovalimab, and in 2 of the 19 patients, they appeared as brief mild to moderate vasculitic cutaneous responses. The two events were resolved while receiving crovalimab treatment [86].

### 8.4. Pozelimab and Cemdisiran as C5 Inhibitors

Pozelimab is under development for PNH treatment (ClinicalTrials.gov identifier: NCT03946748) [87]. A single 15 mg/kg subcutaneous dose of the investigational fully human monoclonal antibody prevented complement-mediated hemolytic activity for at least 35 days [88]. It was shown to bind to both the wild-type and variant (R885H/C) human C5 protein [88]. Complement activation was strongly suppressed in a phase I study in healthy volunteers by frequent high doses of pozelimab (loading dose of pozelimab 15 mg/kg IV followed by four repeat doses of pozelimab 400 mg subcutaneously taken once weekly) [12].

Cemdisiran (phase II clinical trial), an investigational N-acetylgalactosamine-conjugated RNA interference (RNAi) therapeutic that decreases liver C5 production, demonstrated long-lasting suppression of circulating levels of C5 (ClinicalTrials.gov NCT02352493) [89]. However, levels of lactate dehydrogenase (an exploratory goal of the trial) remained above the treatment objective of 1.5 times the upper limit of normal in patients with PNH who were eculizumab-naive, indicating that cemdisiran monotherapy may not be sufficient to provide clinical benefit in PNH [90].

Combining the complementary strategies of C5 reduction and antibody-mediated inhibition may be a better clinical strategy to offer a more patient-friendly infrequent subcutaneous dosing regimen while potentially minimizing intravascular hemolysis breakthroughs. This is because RNAi therapeutics take longer to take effect and do not completely suppress C5; they also require high and frequent dosing due to the target load with anti-C5 antibodies.

The human immunoglobulin G4P monoclonal antibody pozelimab targets C5 and synthetic small interfering RNA cemdisiran targets C5 mRNA. Both medications can be injected subcutaneously. The doses of these two drugs can be significantly decreased by combining them, and the interval for pozelimab dosing can be substantially prolonged, as determined by pharmacokinetic/pharmacodynamic modeling based on data from both pozelimab (NCT03115996) and cemdisiran healthy volunteer research studies (NCT02352493). This represents a promising way of accomplishing clinically significant complement inhibition for prolonged periods [91]. Currently, a phase II clinical trial (NCT04811716) is investigating the tolerability and safety of two-dose regimens for the combination therapy of pozelimab and cemdisiran [92].

### 8.5. Other C5 Inhibitors

A small protein (16 kDa) extracted from the tick *Ornithodoros moubata* is the source of nomacopan, a protein that binds to C5 and prevents C5 convertases from cleaving it [45]. It is being investigated in a long-term phase III clinical trial to assess its efficacy and safety. The findings show that nomacopan provides considerable therapeutic advantages to patients in terms of transfusion independence and is safe and well tolerated for the long-term treatment of PNH by patient self-administration [93].

Tesidolumab, a C5 inhibitor, was effective for PNH patients regardless of non-variant or variant C5, and it had a good safety parameter in a phase II clinical trial. All participants showed significant reductions in their dependence on blood transfusions and in their LDH concentrations, returning them to almost normal levels [94].

### 8.6. Complement Receptor2/Factor H Fusion Protein TT30

A 65-kDa recombinant human fusion protein known as TT30 comprises the inhibitory domain of factor H (fH) and the C3b/C3d-binding region of complement receptor 2 (CR2) [95]. In a modified prolonged acidified serum experiment, TT30 reduced the hemolysis of PNH erythrocytes in a dose-dependent manner and also prevented C3 fragment deposition on surviving PNH erythrocytes [95]. The effectiveness of TT30 arises from its direct interaction with PNH erythrocytes; if this interaction is interrupted, TT30 in solution only partially inhibits hemolysis, similar to the effects of the fH moiety of TT30 alone or of intact human fH [95]. It is possible to stop both the intravascular and C3-mediated extravascular hemolysis of PNH erythrocytes with TT30, which is a membrane-targeted selective CAP (complement alternative pathway) inhibitor [47].

### 8.7. BCX9930, a Factor D Inhibitor

BCX9930 is an oral medication used for the management of diseases mediated by a complement [96]. Factor D, complement system protein, is important in amplifying the responses of the complement system [97]. Factor D inhibition may prevent both intravascular hemolysis and C3-mediated extravascular hemolysis [98]. In a dose-ranging trial, the tolerated doses were up to 500 mg twice daily. Reticulocyte count, hemoglobin and fatigue showed significant improvements in scores from the baseline to week 48, along with concurrent increases in PNH RBC clone sizes [99]. Two phase II clinical trials are underway to evaluate its safety and effectiveness [100].

### 8.8. Danicopan, a Complement Alternative Pathway Factor D Inhibitor

The proven hematological benefit of C5 inhibition may be constrained by the appearance of the C3-mediated emergence of extravascular hemolysis, which cannot be addressed by C5 inhibitors [98]. Danicopan is the first oral proximal factor D inhibitor that blocks this complement alternative pathway [101]. In a phase II dose-ranging, open-label trial, 10 treatment-naive patients with PNH received oral danicopan monotherapy administered at a dose of 100-200 mg thrice daily. Danicopan seemed to be well tolerated and demonstrated clinically significant suppression of intravascular hemolysis and improvement in hemoglobin levels in treatment-naive PNH patients [98]. In the ALPHA phase II trial interim analysis, positive high-level results were found in PNH patients who exhibited significant clinical extravascular hemolysis when danicopan was added to the C5 inhibitor treatment of eculizumab or ravulizumab [102].

### 8.9. ABP 959, an Eculizumab Biosimilar

ABP 959 is a potential biosimilar to eculizumab for the management of PNH and other conditions [103,104]. This monoclonal antibody binds specifically to C5, inhibiting both the classical and alternative complement pathways from progressing [103]. ABP 959 was compared with eculizumab in a randomized, two-period crossover, phase III trial, double-blind, active-controlled DAHLIA study to determine its safety and efficacy in treating adult patients with PNH. No meaningful clinical differences between ABP 959 therapy and eculizumab therapy were observed; immunogenicity and safety profiles were comparable to those of eculizumab [105].

## 9. Conclusions

Understanding the pathophysiology of PNH led to the development of the first complement inhibitor that was authorized for clinical use. C5 inhibition has decreased morbidity and death while improving therapeutic outcomes for patients with PNH. C5 inhibitors, the current gold standard of therapy, target underlying intravascular hemolysis but do not address the residual anemia brought on by insufficient management of intravascular hemolysis in some individuals. C3 deposition on RBCs causes continuous extravascular hemolysis as well as recent cases of hemolysis that have not yet been treated by C5 inhibitors. Patients may still need regular blood transfusions, endure ongoing hemolysis or have persistent chronic anemia. A minor but clinically significant risk of meningococcal infection and headaches is seen in up to one-third of patients treated with eculizumab and ravulizumab. With an 8-week interval between doses, ravulizumab is expected to be a less taxing treatment for patients with PNH and may be linked to a lower incidence of breakthrough hemolysis. Ravulizumab is currently too new to have any long-term evidence to evaluate its safety or effectiveness. Given that ravulizumab contains a black box warning for severe meningococcal infection, long-term safety data are especially crucial.

Additionally, there is little information on whether ravulizumab will be as well tolerated and effective as eculizumab in PNH-affected specific groups, such as pregnant women or young children. Patient responses collected from the study of both eculizumab and ravulizumab indicated a significant burden on quality of life, work productivity, clinical outcomes and healthcare cost resource utilization for patients with PNH. The various treatment options available may have a distinct role in the therapy algorithm (perhaps even one that is personalized to specific patients), which would ultimately result in a significant improvement in the management of PNH.

## Figures and Tables

**Table 1:** Clinical studies of efficacy of eculizumab in patients with PNH.

Number of Patients on Eculizumab	Dose (IV Infusion)	Study Design	Results	References
11	900 mg every 12 to 14 days	Open-label pilot study followed by extension trial	Decrease in LDH, hemolysis, hemoglobinuria, transfusion rate; increase in Type III RBCs	[7,29]
43	600 mg weekly for 4 weeks, followed by 900 mg every other week	Double-blind, randomized, placebo-controlled, multicenter, phase III trial	Reduction in LDH and intravascular hemolysis	[1]
31	600 mg every 7 days for 4 weeks, 900 mg 7 days later and 900 mg every 14 days as a maintenance dose	Non-randomized, comparative study	Low-level hemolysis and nocturnal hemoglobinuria	[30]
79	600 mg every week for 4 doses followed by 900 mg every 14 days	Prospective, single-arm study	Decreased thrombosis and transfusion rate; improved survival rate	[31]
29	600 mg per week for 4 weeks, 900 mg for 1 week and then once every 2 weeks	Open-label, single-arm, multi-center study followed by long-term extension study	Immediate and sustained reduction in intravascular hemolysis and red blood cell transfusions; improvement in fatigue and dyspnea	[2,32]
11	600 mg every 4 weeks followed by 900 mg every other week	Prospective, single-arm study	Reduced the markers of thrombin generation, D-dimers, thrombin anti-thrombin complex (TAT) and inflammation, IL-6	[33]
6	600 mg for 4 weeks followed by 900 mg	Pilot, prospective, open-label, longitudinal clinical study	Decreased extracellular vesicle production and procoagulant profile induced by phospholipids and extracellular vesicles.	[34]
143	600 mg per week for 4 weeks, 900 mg for 1 week and then once every 2 weeks	Long-term extension study	Transfusion independence, reduction in lactate dehydrogenase levels, time-dependent improvement in renal function	[3]

**Table 2:** Potential adverse effects of eculizumab.

Adverse Drug Reaction	Percentage Range of Occurrence	Reference
**Disorders of the musculoskeletal and nervous system**		
Back pain	10–24.6%	[1,3,10]
Arthralgia	7–22.1%	[1,3]
Pain in extremities	3–20%	[3,7]
Myalgia	14.9%	[3]
Lumbar/sacral disc prolapse	2%	[1]
Headache	7–54.9%	[1,2,3,10]
Insomnia	5–20.5%	[2,3,10]
Asthenia	8%	[10]
Fatigue	12–15%	[1,3,10]
Dizziness	5–20%	[1,3]
**Disorders of the digestive system**		
Nausea	16–32.3%	[1,3,7]
Vomiting	5–25.6%	[1,3]
Constipation	14.9%	[3]
Diarrhea	9–34.9%	[1,2,3]
Gastroenteritis	18.5%	[2]
Abdominal pain	5–22.1%	[1,3]
Upper abdominal pain	12.3%	[3]
Cholecystectomies	3.1%	[3]
Elevated blood alkaline phosphatase	11.1%	[2]
**Disorders of the thoracic, respiratory system and mediastinum**		
Pharyngitis	11.1–36.4%	[2,7]
Nasopharyngitis	23–77.8%	[1,2,3]
Upper respiratory tract infection	14–41%	[1,2,3,7]
Oropharyngeal pain	21.5%	[3]
Pneumonia	14.8%	[2]
Flu-like symptom	36.4%	[7]
Cough	12–27.2%	[1,3,7]
Influenza-like illness	17.4%	[3]
Sinusitis	11.8%	[3]
**Disorders of blood, vascular and lymphatic system**		
Contusion	11.1–27.2%	[2,3,7]
Epistaxis	10.8–11.1%	[2,3]
Edema peripheral	10.3%	[3]
Hemolysis	9–23%	[6,7,10]
Neutropenia	6.3–9%	[6,7]
Leucopenia	0–6.3%	[6,7]
Anemia	14.8%	[2]
Thrombotic events (TE) within 8 weeks of the last dose	0.5–2%	[1,3]
Myelodysplastic syndrome	0–1.5%	[3,7,10]
**Immunology**		
Pruritus	10.3%	[3]
Rash	10.3%	[3]
Injection site pain	5–6.3%	[6,10]
Adverse events related to treatment infusion	0–36.4%	[2,3]
Antibody	0	[3,7,10]
**Malignancy**		
Chronic myelomonocytic leukemia	0.5%	[3]
Solid and hematological tumors	2.6 reports per 100 PY	[9]
**Pregnancy**		
Miscarriage	0–15.8%	[3,9]
**Infection**		
Urinary tract infection	0–16.9%	[3,7]
Viral infection	6.3–92.6%	[1,2,3,6]
Aspergillus infection	3%	[3]
Meningococcal infection	0.03–1%	[2,3,9]
Pyrexia	11.8%	[3]
Staphylococcal infection	2%	[1]

**Table 3:** Comparison of adverse effects of eculizumab and ravulizumab.

Adverse Drug Reaction	Percentage Range of Occurrence	Reference
**Disorders in the musculoskeletal and nervous system**		
Fatigue	R = 6.2% E = 6.1%, RR (PEP) = 7.2% (EP) = 13.5% ER (PEP) = 7.1% (EP) = 13.7%	[53,54]
Pain	201 Study R = 0% 103 study R = 7.7%	[27]
Musculoskeletal pain	R = 2.1% E = 5.1%	[53]
Arthralgia	R = 6.4% E = 6.6%, RR (PEP) = 6.4% (EP) = 2.4% ER (PEP) = 6.6% (EP) = 4.2%	[51,52]
Back pain	RR (PEP) = 4.1–6.4% (EP) = 0.8–1.0% ER (PEP) = 4.1–5.0% (EP) = 2.5–6.3%	[52,54]
Pain in extremity	R = 5.2–7.2% E = 4.1–5.8%, RR (PEP) = 5.2–7.2% RR (EP) = 0–4.2% ER (PEP) = 3.1–5.8% (EP) = 2.5–5.3%	[51,52,53,54]
Myalgia	R = 5.6% E = 7.4%, RR (PEP) = 6.4% (EP) = 0.8% ER (PEP) = 7.4% (EP) = 2.5%, 201 study R = 3.8% 103 study R = 0%	[27,51,52]
Insomnia	RR (PEP) = 1.6% (EP) = 2.4% ER (PEP) = 5.0% (EP) = 3.4%	[52]
Dizziness	R = 3.1–7.2% E = 5.8–7.1%, RR (PEP) = 3.1–7.2% (EP) = (0–2.1%) ER (PEP) = 5.8–7.1% (EP) = 0–6.3%	[51,52,53,54]
Headache	R = 26.8–36% E = 17.3–33.1%, RR (PEP) = 27.8–36% (EP) = 4.8–6% ER (PEP) = 19.4–33.1% (EP) = 8.4–10.5%, 201 Study R = 19.2% 302 study R = 7.7%, SC R = 14.1%	[27,51,52,53,54,55]
**Disorders in the digestive system**		
Abnormal GI sounds	201 Study R = 3.8% 302 study R = 0%	[27]
Abdominal pain	R = 6.2% E = 9.2% RR (PEP) = 5.6% (EP) = 2.4% ER (PEP) = 8% (EP) = 5%	[52,53]
Dyspepsia	RR (PEP) = 4% (EP) = 0% ER (PEP) = 5% (EP) = 2.5%	[52]
Vomit	R = 6.2% E = 4.1%	[35]
Nausea	R = 8.2–8.8% E = 8.3–9.2%B RR (PEP) = 8.8% (EP) = 1.65 ER (PEP) = 8.3% (EP) = 5% 201 study R = 3.8% 302 study R = 0%	[27,51,52,53]
Constipation	R = 7.2% E = 5.1%	[51]
Diarrhea	R = 8–9.3% E= 4.1–7.1% RR (PEP) = 8.0–9.3% (EP) = 1.6–6.3% ER (PEP) = 4.1–7.1% (EP) = 3.4–5.3%	[51,52,53,54]
**Disorders of the thoracic, respiratory system and mediastinum**		
Oropharyngeal pain	R = 4.1% E = 9.2% RR (PEP) = 6.4% (EP) = 0% ER (PEP) = 5% (EP) = 0.8%	[52,53]
Cough	R = 5.2% E = 10.2% RR (PEP) = 3.2% (EP) = 1.6% ER (PEP) = 6.6% (EP) = 3.4%	[52,53]
Nasopharyngitis	R = 8.8–21.6% E = 14.9–20.4% RR (PEP) = 8.8–21.6% (EP) = 6.3–6.5% ER (PEP) = 15.7–20.4% (EP) = 7.4–12.6%	[51,52,53,54]
Influenza-like illness	R = 7.2% E = 8.2%	[51]
Rhinitis	R = 5.2% E = 4.1%	[51]
URTI	R = 10.4–18.6% E = 5.8–10.2% RR (PEP) = 10.4–18.6% (EP) = 8.1–9.4% ER (PEP) = 5.8–11.2% (EP) = 4.2–8.4%	[51,52,53,54]
Viral URTI	R = 7.2% E = 8.3% RR (PEP) = 7.2% (EP) = 2.4% ER (PEP) = 8.3% (EP) = 1.7%	[51,52]
**Disorders of the blood, vascular and lymphatic system**		
Atrial flutter	201 study R = 0% 302 study = 7.7%	[27]
TEAE considered a major adverse vascular event	RR (PEP) = 0-1.6% RR (EP) = 0–1% ER (PEP) = 0–0.8% EP = 0.8–1.1%	[52,54]
Thrombophlebitis, superficial	201 study R = 3.8% 302 study R = 0%, R = 1%	[27,54]
Chest pain	R = 3.1% E = 9.2%	[51]
Hypokalemia	RR (PEP) = 4.8% (EP) = 3.2% ER (PEP) = 5% (EP) = 0%	[52]
Neutropenia	201 study R = 3.8% 302 study R = 0%	[27]
Anemia	R = 6.2% E = 3.1% RR (PEP) = 3.2–6.2% (EP) = 0–1% ER (PEP) = 3.1–5% (EP) = 5–5.3% 201 study R = 0% 302 study R = 7.7%	[27,52,53,54]
**Infection and immunology**		
Serious infection	R = 2.1% (influenza and lower respiratory tract infection) E = 1% (acute pyelonephritis)	[51]
Meningococcal infections	201 study R = 7.7% 302 study R = 0% R = 0% E = 0% RR= 0% ER= 0%	[27,51,52,53,54]
Pyrexia	R = 4.8–9.3% E = 5.1–10.7% RR (PEP) = 4.8–9.3% (EP) = 5.6–6.3% ER (PEP) = 5.1%–10.7% (EP) = 0–6.3% SC R = 10.9%	[51,52,53,54,55]
Antidrug antibody	R = 0–0.8 E = 0–0.8 RR= 0, ER= 0	[27,51,52,53,54]
Infusion site reaction	201 study R = 3.8% 302 study R = 0% R SC = 4.7%	[27,55]

PEP—Primary evaluation period, EP—Extension period, sc—subcutaneous, E—Eculizumab, R—Ravulizumab.

## Supplementary Materials

The following supporting information can be downloaded at: https://www.mdpi.com/article/10.3390/medicina60030379/s1. Supplementary File S1: Search terms.
