# Supplementary material for: Safety Profile of Monoclonal Antibodies and Subsequent Drug Developments in the Treatment of Paroxysmal Nocturnal Hemoglobinuria
Source: Medicina (Kaunas). 2024 Feb 24;60(3):379. doi: 10.3390/medicina60030379 (PMC10971871; doi:10.3390/medicina60030379)
Supplement: Supplementary file 1 [file medicina-60-00379-s001.zip › medicina-2776227-supplementary.pdf]

Supplementary file 1

Literature search was conducted in Pubmed, Embase, and Web of science.

Pubmed

#1 #2 #3 AND #4

#1

("PNH"[All Fields] OR ("hemoglobinuria, paroxysmal"[MeSH Terms] OR ("hemoglobinuria"[All Fields] AND "paroxysmal"[All Fields]) OR "paroxysmal hemoglobinuria"[All Fields] OR ("paroxysmal"[All Fields] AND "nocturnal"[All Fields] AND "hemoglobinuria"[All Fields]) OR "paroxysmal nocturnal hemoglobinuria"[All Fields])) AND "patho*"[All Fields]

#2

(("PNH"[All Fields] OR ("hemoglobinuria, paroxysmal"[MeSH Terms] OR ("hemoglobinuria"[All Fields] AND "paroxysmal"[All Fields]) OR "paroxysmal hemoglobinuria"[All Fields] OR ("paroxysmal"[All Fields] AND "nocturnal"[All Fields] AND "hemoglobinuria"[All Fields]) OR "paroxysmal nocturnal hemoglobinuria"[All Fields])) AND ("therapeutics"[MeSH Terms] OR "therapeutics"[All Fields] OR "treatments"[All Fields] OR "therapy"[MeSH Subheading] OR "therapy"[All Fields] OR "treatment"[All Fields] OR "treatment s"[All Fields])) OR (("monoclonal"[All Fields] OR "monoclonality"[All Fields] OR "monoclonally"[All Fields] OR "monoclonals"[All Fields] OR "monoclone"[All Fields] OR "monoclones"[All Fields]) AND "antibody*"[All Fields]) OR ("efficacies"[All Fields] OR "efficacious"[All Fields] OR "efficaciously"[All Fields] OR "efficaciousness"[All Fields] OR "efficacy"[All Fields])

#3

("ravulizumab"[Supplementary Concept] OR "ravulizumab"[All Fields]) AND ("eculizumab"[Supplementary Concept] OR "eculizumab"[All Fields]) AND ("safety"[MeSH Terms] OR "safety"[All Fields] OR "safeties"[All Fields]) AND ("PNH"[All Fields] OR ("hemoglobinuria, paroxysmal"[MeSH Terms] OR ("hemoglobinuria"[All Fields] AND "paroxysmal"[All Fields]) OR "paroxysmal hemoglobinuria"[All Fields] OR ("paroxysmal"[All Fields] AND "nocturnal"[All Fields] AND "hemoglobinuria"[All Fields]) OR "paroxysmal nocturnal hemoglobinuria"[All Fields]))

#4

("PNH"[All Fields] OR ("hemoglobinuria, paroxysmal"[MeSH Terms] OR ("hemoglobinuria"[All Fields] AND "paroxysmal"[All Fields]) OR "paroxysmal hemoglobinuria"[All Fields] OR ("paroxysmal"[All Fields] AND "nocturnal"[All Fields] AND "hemoglobinuria"[All Fields]) OR "paroxysmal nocturnal hemoglobinuria"[All Fields])) AND ("therapeutics"[MeSH Terms] OR "therapeutics"[All Fields] OR "treatments"[All Fields] OR "therapy"[MeSH Subheading] OR "therapy"[All Fields] OR "treatment"[All Fields] OR "treatment s"[All Fields]) AND ("new"[All Fields] OR "newer"[All Fields] OR ("pipeline"[All Fields] OR "pipeline s"[All Fields] OR "pipelined"[All Fields] OR "pipelines"[All Fields] OR "pipelining"[All Fields]))

Embase

(('paroxysmal nocturnal hemoglobinuria'/exp OR 'paroxysmal nocturnal hemoglobinuria') AND patho* OR (pnh AND (treatment OR drug)) OR (pnh AND monoclonal AND antibody AND (safety OR efficacy)) OR (pnh AND (difficulty OR challenge) AND (treatment OR drug)) OR (pnh AND (new* OR pipeline) AND (treatment OR drug))) AND ('animal tissue'/de OR 'case control study'/de OR 'case report'/de OR 'case study'/de OR 'cell culture'/de OR 'clinical article'/de OR 'clinical study'/de OR 'clinical trial'/de OR 'clinical trial topic'/de OR 'cohort analysis'/de OR 'comparative effectiveness'/de OR 'comparative study'/de OR 'controlled clinical trial'/de OR 'controlled study'/de OR 'cross sectional study'/de OR 'diagnostic test accuracy study'/de OR 'disease model'/de OR 'double blind procedure'/de OR 'ex vivo study'/de OR 'human'/de OR 'human cell'/de OR 'human tissue'/de OR 'in vitro study'/de OR 'in vivo study'/de OR 'major clinical study'/de OR 'medical record review'/de OR 'methodology'/de OR 'model'/de OR 'multicenter study'/de OR 'normal human'/de OR 'observational study'/de OR 'open study'/de OR 'phase 1 clinical trial'/de OR 'phase 1 clinical trial topic'/de OR 'phase 2 clinical trial'/de OR 'phase 2 clinical trial topic'/de OR 'phase 3 clinical trial'/de OR 'phase 3 clinical trial topic'/de OR 'pilot study'/de OR 'practice guideline'/de OR 'prospective study'/de OR 'randomized controlled trial'/de OR 'randomized controlled trial topic'/de OR 'retrospective study'/de) AND 'article'/it

Web of science

#1 OR #2 OR #3 OR #4 OR #5 and Article (Document Types) and English (Languages)

#1

((ALL=(Paroxysmal nocturnal haemoglobinuria)) OR ALL=(PNH)) AND ALL=(patho*)

#2

(((ALL=(PNH)) OR ALL=(paroxysmal nocturnal haemoglobinuria)) AND ALL=(treatment)) OR ALL=(drug)

#3

(((ALL=(PNH)) OR ALL=(paroxysmal nocturnal haemoglobinuria)) AND (ALL=(monoclonal antibody)) OR ALL=(ravulizumab) OR ALL=(eculizumab)) AND (ALL=(safety))

#4

(((ALL=(PNH)) OR ALL=(paroxysmal nocturnal haemoglobinuria)) AND (ALL=(treatment) OR ALL=(drug)) AND (ALL=(difficulty) OR ALL=(challenge)))

#5

((ALL=(PNH) OR ALL=(paroxysmal nocturnal haemoglobinuria)) AND (ALL=(new*) OR ALL=(pipeline)) AND (ALL=(treatment) OR ALL=(drug)))
